# Extensive systemic thrombo-embolism including intra-cardiac thrombosis mimicking an atrial myxoma in a patient with beta thalassaemia major – a case report

**DOI:** 10.1186/s12872-023-03576-2

**Published:** 2023-10-31

**Authors:** Chiranthi Welhenge, Rumesh Ranasinghe, Sanjeewa Rajapakse, Anuja Premawardhena

**Affiliations:** 1 University Medical Unit, North Colombo (Teaching) Hospital, Ragama, Sri Lanka; 2 Cardiology Unit, North Colombo (Teaching) Hospital, Ragama, Sri Lanka; 3 Department of Medicine, Faculty of Medicine, University of Kelaniya, Ragama, Sri Lanka

**Keywords:** Thalassemia major, Splenectomy, Sepsis, Iron overload, Thrombo-embolism, Case report

## Abstract

**Background:**

Sepsis and thrombo-embolic disease are well known complications of thalassemia major. Intracardiac thrombi are however rare and can lead to diagnostic dilemmas.

**Case presentation:**

We report the case of a 20-year-old female splenectomised thalassaemia major patient with severe iron overload, who presented with life threatening sepsis associated with a liver abscess. Discovery of a large oscillating intra cardiac lesion on 2D echocardiogram confirmed by Contrast Enhanced Computed Tomography (CECT) chest in the right atrium extending from the left hepatic vein through the inferior vena cava complicated the clinical course. After a prolonged Intensive Care Unit (ICU) stay supported with antibiotics and anticoagulation, she recovered with evidence of resolution of the intra cardiac thrombus.

**Conclusions:**

Early recognition and prompt aggressive treatment of sepsis in patients with thalassemia is essential to prevent complications. Intracardiac thrombosis is a potentially treatable cause for an intra cardiac mass in patients with thalassemia major, which should not be missed.

**Supplementary Information:**

The online version contains supplementary material available at 10.1186/s12872-023-03576-2.

## Introduction

Survival of Thalassaemia major (TM) patients is steadily increasing in many parts of the world and is directly related to the standard of care. Median survival of TM patients in Italy, Cyprus and Greece ranges from 41 to 54 years with 7% of patients in the 55—64 age group in Italy and 19% of patients more than 61 years in Greece [1–5]. These encouraging values are not seen in South Asian countries where most patients are not expected to live past the second decade [6]. Commonest causes of death in TM remains heart failure due to iron over-load, but sepsis too is an important cause [7]. Infections including abscess formation in different sites is well known in patients with thalassemia [8]. Another serious complication leading to morbidity and mortality in patients with thalassaemia is thrombo-embolic disease [9]. Here we report a case history of a patient with TM who had severe iron overload, liver abscess together with extensive venous thrombo-embolism, including intra cardiac thrombosis, which is a rare occurrence in TM, despite the high incidence of thrombo-embolic disease.

## Case history

Miss. S, 20-year-old splenectomised patient with beta thalassaemia major was in hospital for the investigation of a three-day history of fever and non-specific bilateral shoulder joint pain of two days duration. Clinically she was not febrile but was ill and carried a C-Reactive Protein (CRP) report done through self-referral reading 240 (normal < 5). Initial clinical assessment could not identify the cause for the fever or the markedly elevated CRP. 16 h later she became confused and developed shortness of breath and her blood pressure dropped to 80/40 mmHg. She was resuscitated and was transferred to the Intensive Care Unit (ICU) for further care. Her investigations revealed high leukocyte counts with neutrophil leukocytosis, persistently elevated CRP levels and deranged liver enzymes. She had a prolonged ICU stay where she was treated for a suspected “lower respiratory tract infection” with multiple combinations of antibiotics including IV Ceftazidime, Piperacillin/Tazobactum, Amikacin, Teicoplanin, Meropenum, Ciprofloxacin and Doxycycline over the duration under the guidance of a Consultant Microbiologist. The clinical response remained poor as did the response of hypoxia which warranted long periods of intermittent Continuous Positive Airway Pressure (CPAP) ventilation. Initial investigations including a trans thoracic echocardiogram did not show significant cardiac abnormalities. Sixteen days into the illness, Contrast Enhanced Computed Tomography (CECT) chest abdomen revealed the presence of a liver abscess, 5 cm × 3.9 cm, in the left lobe of the liver with left hepatic vein thrombosis and multiple septic emboli in the lungs. A repeat echocardiogram at this stage revealed a 3.6 cm oscillating mass in the right atrium, suspected to be a thrombus or an atrial myxoma. Table 1 shows the summary of investigations through the course of the illness.

**Table 1:** Summary of investigations through the course of illness

	D3	D4	D8	D11	D13	D15	D17	D19	D21	D23	D25	D30	D39	D51
WBC	15.5	24	50.7	66.4	40.5	31.3	34.97	33	25.5	17.4	18.6	11.5	23.7	24.24
PLT	124	126	326	34	30	83	212	120	65		6	29	186	374
CRP	282	306	232	315	178	143	149	67.7	31.3	52.4		18.9	4.8	
ALT	87	107	61	175	162	62	31	21	19	23		42	29	37
AST	101	110	55	654	142	38	37	37	54	35		50	59	67
ALP	290	184		381				213		183		327	317	238
T. Bil	21.4	26.8		40.4	26.1			23.3		24.5		29.5	19.6	20.3
D.Bil	16.6	25.7		33.9	19.3			12.6		14		22.9	10.1	11.9
INR		1.27		2.17		1.3	1.3	1.27	1.3					1.37
Blood culture		Negative		Negative	Negative			Positive						
2D ECHO		Normal	Normal					Atrial mass						

**Fig. 1:**
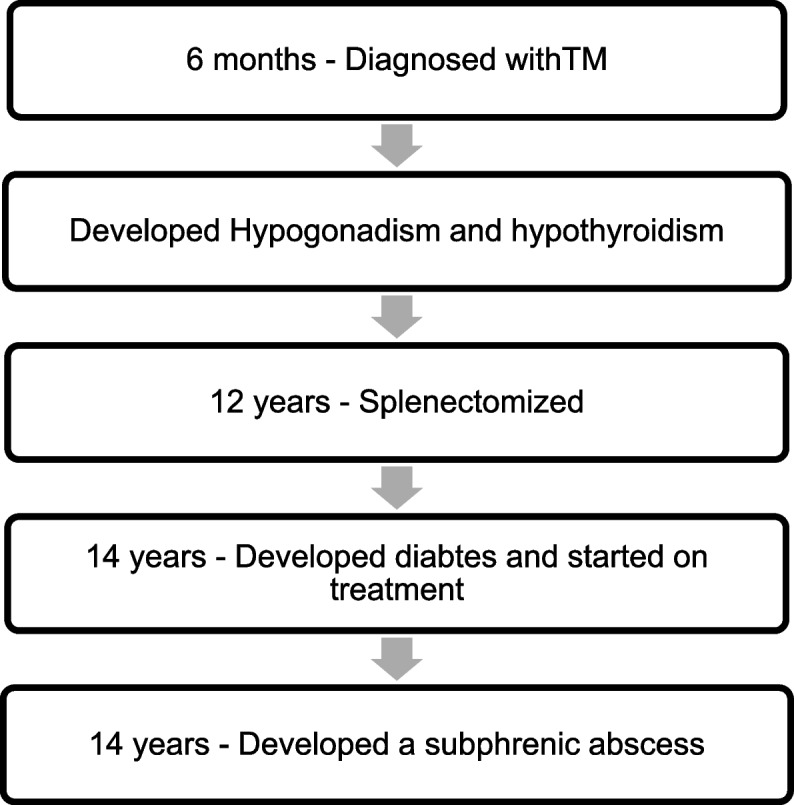
Timeline of significant past events

Beta thalassemia major had been diagnosed at the age of 6 months, and she had been on monthly blood transfusions since. Her mean pre transfusion Haemoglobin (Hb) level over the last year was 9 g/dl. There was evidence of severe iron overload with a mean ferritin value of more than 2000 ng/ml and a highest recorded ferritin value of 13 649 ng/ml. Her compliance to the chelators had been inconsistent. She had developed hypogonadism but was not on hormone replacement therapy, hypothyroidism but was not on thyroxine and had developed diabetes mellitus for which insulin had been started 6 years ago. She has undergone splenectomy at the age of 12 years and was on oral penicillin prophylaxis. She had received one dose of Pneumococcal and Meningococcal vaccines and three doses of Hepatitis B vaccines prior to splenectomy in 2011 but has not received any booster doses of the pneumococcal vaccine. She has had one previous severe infection; a sub phrenic abscess in 2016, which had resolved with one month of in-hospital treatment. Figure 1 shows the timeline of significant past events.

During the present admission, her repeat blood cultures available on day 18 of the illness, grew a Vancomycin resistant enterococcus which was sensitive only to Linezolid. Melioidosis antibodies and Human Immunodeficiency Virus (HIV) screening had been negative. Clinical and inflammatory marker improvement was quick with the introduction of intravenous Linazolid. This was supported by ultrasound scan evidence of resolution of the liver abscess 2 weeks later. Anticoagulation for the hepatic venous thrombosis had to be delayed as she developed severe thrombocytopenia with features of microangiopathic haemolysis due to sepsis, the slow recovery of which was attributed to Linazolid. Once Linazolid was stopped the platelet count started to improve and anticoagulation was commenced.

One week after starting anticoagulation she underwent a repeat CECT which revealed the presence of a resolving liver abscess but left hepatic vein thrombosis was seen extending through the inferior vena cava in- to the right atrium without significant interval change in size and extension. There was resolution of all septic emboli in the lungs except for one. A cardiothoracic surgical opinion was sought due to the high risk of thrombo-embolism posed by the right atrial thrombus/myxoma. It was decided not to explore the right atrium surgically, but to continue to offer life-long anticoagulation with follow up echocardiograms, considering the high risk of the surgery. The patient and the family also refused surgical interventions and her autonomy was respected. She agreed to continue lifelong anticoagulation. Meanwhile her iron chelation was optimized. On discharge her serum ferritin level was 1800 ng/ml. Follow up echocardiogram two months later revealed good resolution of the thrombus while on oral anticoagulation.

## Discussion

Patients with TM are at risk of severe sepsis due to various reasons. The causes include the abnormalities in the immune system related to the disease including defective T and B lymphocyte function as well as the complement system. Patients with high iron levels are vulnerable to infections with siderophillic organisms such as *Klebsiella* spp, *Yersinia enterocoloitica*, E.*coli*, *Vibrio vulnificus*, *Streptococcus pneumoniae*, *Listeria monocytogenes*, *Pseudomonas aeruginosa* and *Legionella pneumophila*. Recurrent blood transfusions also predispose to blood borne infections. Iron chelation therapy also predispose to infections such as *Yersinia enterocolitica*. Splenectomised patients with TM are at risk of severe sepsis due to capsulated organisms especially if proper vaccination procedures are not undertaken [10]. Diabetes which occurs as a complication of iron overload further suppresses the immune system of these patients pre-disposing them to invasive infections. The spectrum of diseases related to sepsis in TM patients ranges from simple viral infections to abscesses at different places to disseminated sepsis including infective endocarditis [8, 11–16].

Our patient had multiple risk factors which pre-disposed her to sepsis. She was splenectomised and had the vaccinations prior to her surgery without any booster doses and had poor compliance to antibiotic prophylaxis. She also had severe iron overload which was not managed adequately due to poor compliance of the patient as well as inadequate monitoring from her health care services. Brittle diabetes with poor glycemic control also contributed further to her immunosuppression. As a patient subjected to multiple and difficult cannulations for regular blood transfusions this is likely to introduce infections unless strict aseptic procedures are adhered to.

The initial non-specific symptoms with sudden onset respiratory distress and high inflammatory markers along with deranged liver enzymes led the clinical team to think of a respiratory pathology as the primary focus of infection. However, the persistence of high swinging fever spikes despite minimal lung signs while on multiple broad-spectrum antibiotics made it evident that the focus of infection was not the lung. Since the ultrasound scan of the abdomen and the 2D echocardiogram repeatedly became normal it was a diagnostic dilemma to the clinical team which led them to arrange a CECT chest abdomen pelvis which ultimately revealed the presence of the liver abscess with multiple septic emboli in lungs and thrombosis of the hepatic vein. This highlights the importance of early and repeated utilization of imaging investigations in patients with immunosuppression for identification of the focus of the infection which could ultimately lead to early treatment.

The presence of multiple septic foci warranted a differential diagnosis of severe disseminated sepsis, infective endocarditis or melioidosis. The 2D echocardiogram at this stage revealed the presence of a large oscillating mass in the right atrium, which was unlikely to be a vegetation, considering its large size. The repeat blood cultures at this point became positive for Vancomycin resistant enterococci which was sensitive only to Linezolid and patient became negative for melioidosis. This signifies the importance of repeating relevant investigations in situations when the clinical picture does not tally with the available investigations.

The identity of the right atrial mass remained a conundrum for weeks since the patient was not suitable to undergo a Trans Oesphageal Echocardiogram (TOE) at that time. The possibility of a thrombus or a myxoma was high on the list of differential diagnoses considering the size of the mass. Considering that all her past echocardiograms including the one done on admission were normal, the possibility of a myxoma was considered unlikely since it was highly unlikely for a cardiac mass to grow rapidly to reach a size like this. An increased incidence of myxomas in patients with TM was not known and rapid growth of a myxoma had been reported only once which had been attributed to severe immunosuppression [17]. Thalassaemia is well recognized to be a hypercoagulable state [8] Since TM patients are at high risk of thrombo-embolic disease the chance of the mass being a thrombus was considered to be more likely [18]. However, the prevalence intra cardiac thrombosis in patients with TM is extremely rare [19].

**Fig. 2:**
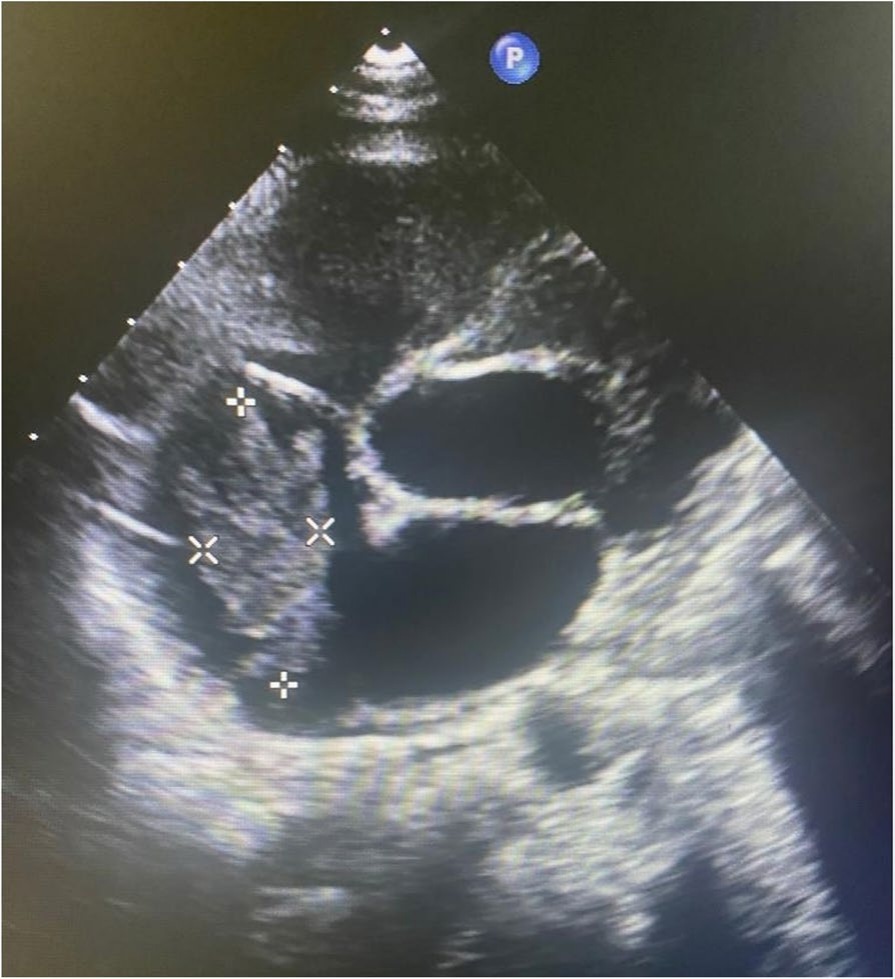
Right atrial mass with a stalk

Our patient underwent a TOE subsequently which revealed the presence of an oscillating mass attached to right atrium via a stalk – Fig. 2. The repeat CECT done revealed the presence of hepatic vein thrombosis extending through the inferior vena cava into the right atrium without significant interval change compared with the previous CECT. We concluded the right atrial mass to be a thrombus which would have become infected giving rise to multiple septic emboli which lead to severe consequences in this patient. A literature survey revealed only one similar case report where intra cardiac thrombosis was found in a splenectomized patient with TM [20]. Intracardiac thrombosis in relation to sepsis in TM is rarely reported, which makes our case unique.

Through this case report we wish to highlight the significance of sepsis probably acting as a precipitant for venous thrombosis, presenting as an intra-cardiac thrombosis in a patient with thalassaemia major.

## Conclusion

Patients with TM are at high risk of severe sepsis due to multiple reasons. Early thorough investigations are required for the early identification of the focus of infection and early treatment. Although thrombo-embolic disease is well known in TM, intracardiac thrombosis is not frequently reported. However, it should not be missed and should be considered as one of the most important differentials in a patient with an intracardiac mass.

### Supplementary Information

**Additional file 1.** 

**Additional file 2.** 

## Data Availability

This case report did not involve collection of data. However, patient’s clinical data is available from the Bed head ticket which can be retrieved. The dataset(s) supporting the conclusions of this article is(are) included within the article (and its additional file(s)).
